# Thoracoscopic resection of paraesophageal ectopic mediastinal parathyroid adenoma in the superior posterior mediastinum: a case report

**DOI:** 10.1186/s13019-024-02729-4

**Published:** 2024-04-16

**Authors:** Kai Fu, Xin-Yu Zhu, Xin-Yu Jia, Kun-Peng Feng, Yuan Cui, Jun Zhao, Chang Li

**Affiliations:** 1 Department of Thoracic Surgery, The First Affiliated Hospital of Soochow University, Medical College of Soochow University, Suzhou, 215000 China; 2 Institute of Thoracic Surgery, The First Affiliated Hospital of Soochow University, Suzhou, 215000 China

**Keywords:** Paraesophageal, Ectopic parathyroid, Video-assisted thoracoscopy, Hyperparathyroidism

## Abstract

**Background:**

The ectopic superior parathyroid in the tracheoesophageal groove and paraesophageal region is rare. Hyperparathyroidism results when these glands become hyperfunctioning. That may necessitate surgical intervention in the form of parathyroidectomy, which requires a transsternal or transthoracic approach due to a deeply seated mediastinal parathyroid gland. Minimally invasive strategies have emerged recently as an alternative approach with less morbidity.

**Case presentation:**

We present a case of the paraesophageal ectopic parathyroid gland in the superior posterior mediastinum, which was successfully treated with thoracoscopic resection.

**Conclusion:**

The current imaging tools improve the thoracoscopic management of mediastinal parathyroid glands. Video-assisted thoracoscopic surgery (VATS) can provide access and exposure to ectopic parathyroid adenoma with low morbidity and financial burden.

## Background

The parathyroid glands originate from the endoderm and develop from the dorsal wing of the 3rd and 4th pharyngeal pouches [1]. Primary hyperparathyroidism is characterized by excessive secretion of parathyroid hormone due to parathyroid adenoma, hyperplasia, or rarely parathyroid cancer. Asymptomatic presentation is not uncommon [2]. Most abnormal parathyroid glands are found in the superior mediastinum within the thymus and can be removed through a cervical incision which is challenging if the adenoma is seated deeply in the mediastinum, and typically require a thoracotomy. Due to recent advances in video-assisted thoracoscopic surgery and accurate preoperative localization, minimally invasive surgery is now the preferred method. In this case, we describe our successful video-assisted approach to the resection of a paraesophageal ectopic parathyroid gland in the superior posterior mediastinum.

## Case presentation

**Fig. 1:**
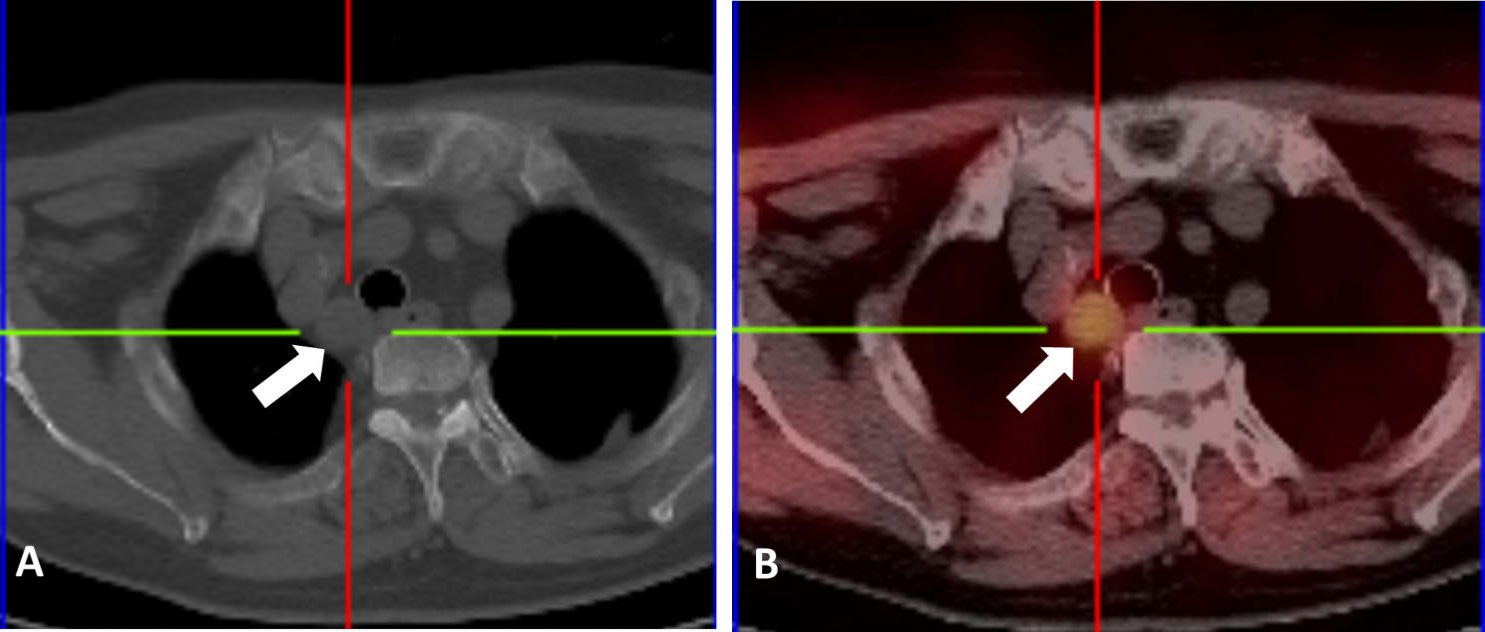
Patient imaging. (**A**) Contrast-enhanced computed tomography of the chest demonstrating a 2.4x1.4cm solid posterior mediastinal mass with contrast enhanced in the edge abutting the trachea and esophagus(arrow). (**B**) 99mTechnetiummethoxy-isobutyl-isonitrite (MIBI) scintigraphy demonstrating a large distinct accumulation of radiotracer in the right posterior superior mediastinum(arrow)

**Fig. 2:**
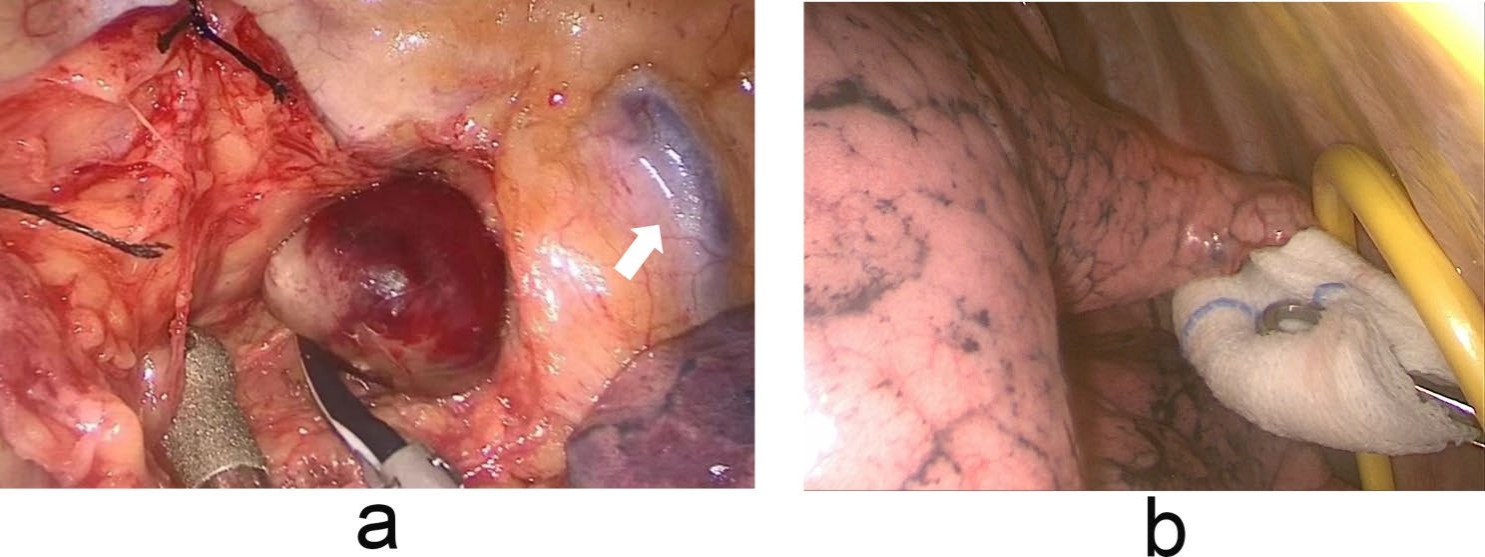
Operative imaging. (**A**) Posterior mediastinal mass is seen abutting the right innominate vein(arrow). (**B**) The operative port was placed in the right midaxillary line in the fourth intercostal space(Yellow protective ring)

**Fig. 3:**
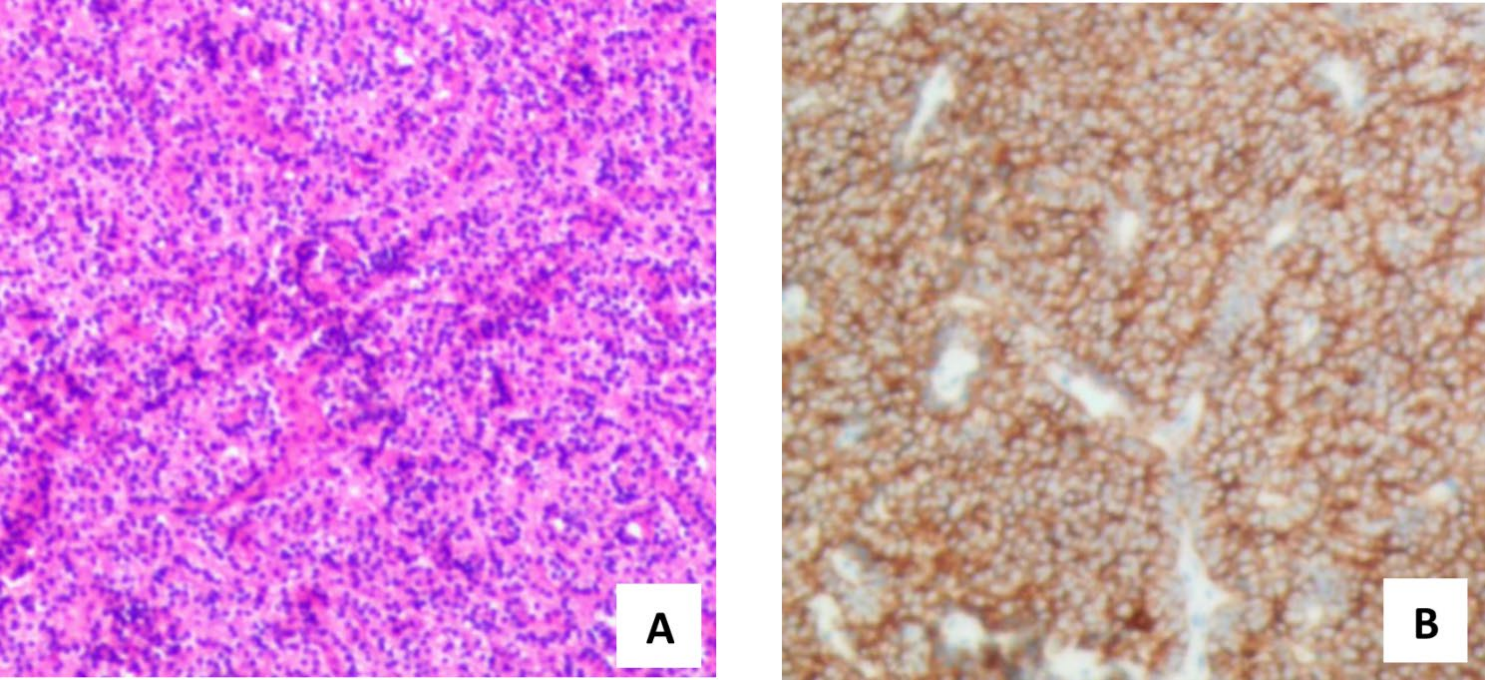
. Final pathologic assessment. (**A**) trabeculae of parathyroid chief cells (arrows). (**B**) Staining for parathyroid hormone

A 66-year-old woman was found to have hypercalcemia during a physical examination. The patient with a 10-year history of hypertension had no familial history of multiple endocrine neoplasia and denied any allergies. Blood biochemical examination demonstrated an elevated serum calcium level of 3.21mmol/L (normal range, 2.11-2.52mmol/L) and a diminished serum phosphorus level of 0.83mmol/L (normal range, 0.85-1.51mmol/L). Contrast-enhanced computed tomography of the neck and thorax revealed a 24 × 14 mm solid mass in the superior posterior mediastinum. The lesion showed contrast enhancement in the edge (Fig. 1A). 99mTechnetium methoxy-isobutyl-isonitrite (MIBI) scintigraphy disclosed an area of high tracer uptake in the posterior-superior mediastinal (Fig. 1B). Consequently, the patient was taken to the operating room for a right thoracoscopic resection of the mediastinal mass. The patient was placed in the left lateral position. A total of two ports which were marked on the skin were placed. The location of the operative port (3 cm) was placed in the right midaxillary line in the fourth intercostal space. Having regard to the location of the operative field in the superior posterior mediastinum, we decided to set the placement of our viewing port (1.5 cm) to the seventh intercostal space of the posterior axillary line. A solid structure was visualized arising from the posterior mediastinum and abutting the right innominate vein (Fig. 2). Pulling adipose tissue on the side was required to facilitate dissection and removal of the mass completely. The operation was successful and the intraoperative blood loss was less than 50 ml. A chest drain was inserted. The postoperative pathology (Fig. 3) confirmed the diagnosis of ectopic parathyroid adenoma. Additionally, immunohistochemical staining for CgA and CK was positive. The serum calcium levels (2.30mmol/L on the first postoperative day; normal range, 2.11 to 2.52mmol/L) decreased to normal level dramatically. There were no postoperative complications related to bleeding and air leakage. The mediastinal drainage tube was removed on the second postoperative day and the patient was discharged home on the same day in stable condition. Then the patient returned for follow-up 2 weeks later, where she remained asymptomatic with a normal calcium of 2.21mmol/L.

## Discussion

Parathyroid adenomas resulted in nearly 80% of all primary hyperparathyroidisms which weigh heavily for hypercalcemia. Abnormal, hypersecreting parathyroid glands are found in ectopic locations in up to 15–20% of patients [3].

Imaging figures prominently in the preoperative evaluation, especially for asymptomatic patients. Minimally invasive surgery relies on accurate localization of ectopic parathyroid adenomas which can increase the success rate of treatment and decrease the recurrence rate. Ultrasonogram diagnosis, Computed tomography (CT), and other imaging tools are used for diagnosis and localization.

Surgery is the definitive treatment of choice to control the morbidity associated with primary hyperparathyroidism. The paraesophageal glands locating in the superior posterior mediastinum account for 5–10% of ectopic glands, and are challenging to thoracoscopic resection or even open thoracotomy. VATS is associated with shorter surgical time and less intraoperative bleeding for patients compared to thoracotomy approaches [4]. Ismail et al. reported the use of robotic surgery for the resection of parathyroids in the anterior mediastinum was feasible and effective [5]. There are few reports related to thoracoscopic resection of paraesophageal ectopic parathyroids in the posterior superior mediastinum.

In our case, our experience shows that a double-port thoracoscopic approach for paraesophageal parathyroids in the deep superior mediastinum is safe and effective, and has the advantages of facilitating recovery quickly and economy.

## Conclusion

When paraesophageal ectopic parathyroids in the posterior superior mediastinum are detected preoperatively with localization tools, VATS is a safe, economical and effective approach.

## Data Availability

No datasets were generated or analysed during the current study.

## Abbreviations

MIBI: 99mTechnetium methoxy-isobutyl-isonitrite
CT: Computed tomography
VATS: video-assisted thoracoscopic surgery
